# Correction of Inverted Nipple Using Subcutaneous Turn-Over Flaps to Create a Tent Suspension-Like Effect

**DOI:** 10.1371/journal.pone.0133588

**Published:** 2015-07-24

**Authors:** Hii-Sun Jeong, Hye-Kyung Lee

**Affiliations:** 1 Department of Plastic & Reconstructive Surgery, Kangnam Sacred Heart Hospital, Hallym University Medical Center, Hallym University College of Medicine, Seoul, Republic of Korea; 2 Department of Plastic and Reconstructive Surgery, Eulji General Hospital, Eulji University School of Medicine, Seoul, Republic of Korea; University of Salerno, ITALY

## Abstract

**Background:**

Many techniques have been reported for the correction of inverted nipples. However, the conventional methods may be insufficient, especially for moderate to severe inversions. We propose a modification of Elsahy’s method and report satisfactory results.

**Methods:**

A single-institutional retrospective review was performed for all patients who received the modified operation. Patient charts were reviewed for demographic data, pertinent preoperative factors such as Han and Hong classification, and clinical outcomes including postoperative nipple height and sensation. Surgical details are described within the main text.

**Results:**

The review identified 26 female patients amongst whom 47 inverted nipples were corrected using the modified method. The mean nipple height was 9 mm with an average follow-up period of 14 months. Brush stimulation elicited nipple contraction in all patients. There was no recurrence of nipple inversion, nor were there any surgical complications to report.

**Conclusion:**

The suspension technique is a simple, reliable method for correcting grade II and III nipple inversions.

## Introduction

An inverted nipple is a condition in which a portion of or the entire nipple is buried below the plane of the areola. The condition was first described by Cooper in 1840, and the first corrective operation was reported by Kehrer in 1879. In patients with inverted nipple, a relatively short lactiferous duct is attached to the nipple via dense and highly inelastic connective fibers [1, 2]. This deformity can pose aesthetic, psychological, and functional problems such as difficulty with breastfeeding.

Nipple inversion is not rare, with reported prevalence ranging from 1.8 to 3.3% [1, 3]. Over time, a number of methods have been used to correct the condition. Elsahy originally proposed the use of bilateral triangular dermal flaps that cross under the nipple [4, 5]. Due to its simplicity and effectiveness, this method came to be used widely both in its original and modified forms [6–11].

Despite the variety in corrective operations, postoperative recurrence of inversion remains a problem. In the current study, we introduce a modified operation with rotated-buried flaps and report clinical outcomes of this technique, along with a review of the literature.

## Patients and Methods

All research involving human participants had been approved by the institutional review board at Myong-Ji Hospital (Goyang, South Korea). All of the patient's records and information was anonymized and de-identified prior to analysis. Additionally, all patients whose clinical photographs have been included in this article gave written approval of the use of their photographs for research, presentation, and publication.

The retrospective study was performed for all patients undergoing the described inverted nipple correction from 1999 to 2010. Medical charts of identified patients were reviewed for demographic data, pertinent preoperative factors such as Han and Hong classification, and clinical outcomes including postoperative nipple height and sensation. Additionally, charts were reviewed for complications such as nipple necrosis, permanent numbness, hematoma, infection, or breastfeeding difficulty.

### Surgical technique

Operations were performed under topical and local anesthetic. Upon surgical preparation, a modified Elsahy incision was designed over inverted nipple. The modification was such that the triangular flaps had wider bases (each ¼ of the circumference of nipple base) with flap lengths equal to the nipple diameter (Fig 1). The nipple was pulled anteriorly with a 5–0 nylon stay suture, and the nipple base was circumferentially incised to the superficial dermis.

**Fig 1:**
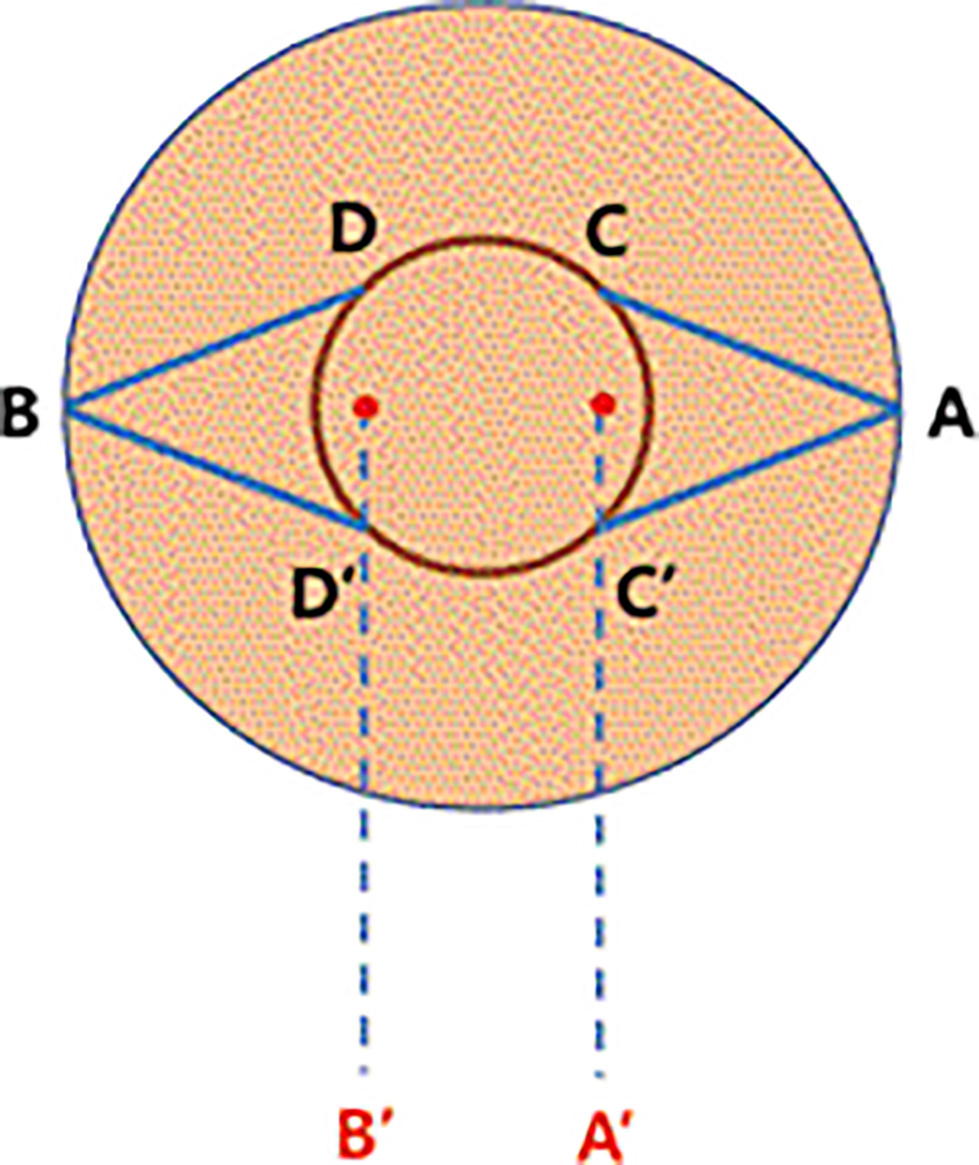
A schematic diagram of the design of the method for inverted nipple correction. Full-thickness subcutaneous triangular flaps are designed as shown in the diagram after pulling out the inverted nipple. The red dotted circle means the base of the nipple before traction. B’ is the dermal point of approximation of D and D’ from the areola, and A’ is the dermal point of approximation of C and C’ from the areola. After traction of the inverted nipple, the circular area of the nipple base becomes larger. The long axis of the triangular flap is equal to the diameter of the nipple base (BB’ = B’A’ = A’A). The tip of the triangular flap, or A, is overturned and fixated to B’, which is the dermal point to which D and D’ of the areola are approximated. The tip of the opposite triangular flap, B, is then fixated to A’, to which C and C’ are approximated.

Each of the triangular flaps were de-epithelialized and elevated to include the areolomammillary muscle layer and subcutaneous tissue just above the breast parenchyma (Fig 2). With the nipple under gentle traction, a vertical tunnel was created deep to the central portion of the nipple. This dissection was carried out bluntly to avoid injury to the lactiferous duct (Fig 3). The triangular flaps were rotated about the axis and downturned into this pocket such that the deeper surfaces of the flaps were in contact with each other, while the superficial surfaces were in touch with the walls of the dissection pocket (Fig 4). The flaps were fixed to each other at three of the respective vertices with 5–0 polyglactin sutures. The subcutaneous layers were approximated with 5–0 polyglactin, and the areola skin was closed with 6–0 nylon. The nipple base was then re-draped to adjust for the tension from twisting of the triangular flaps and primary closure of the areola skin.

**Fig 2:**
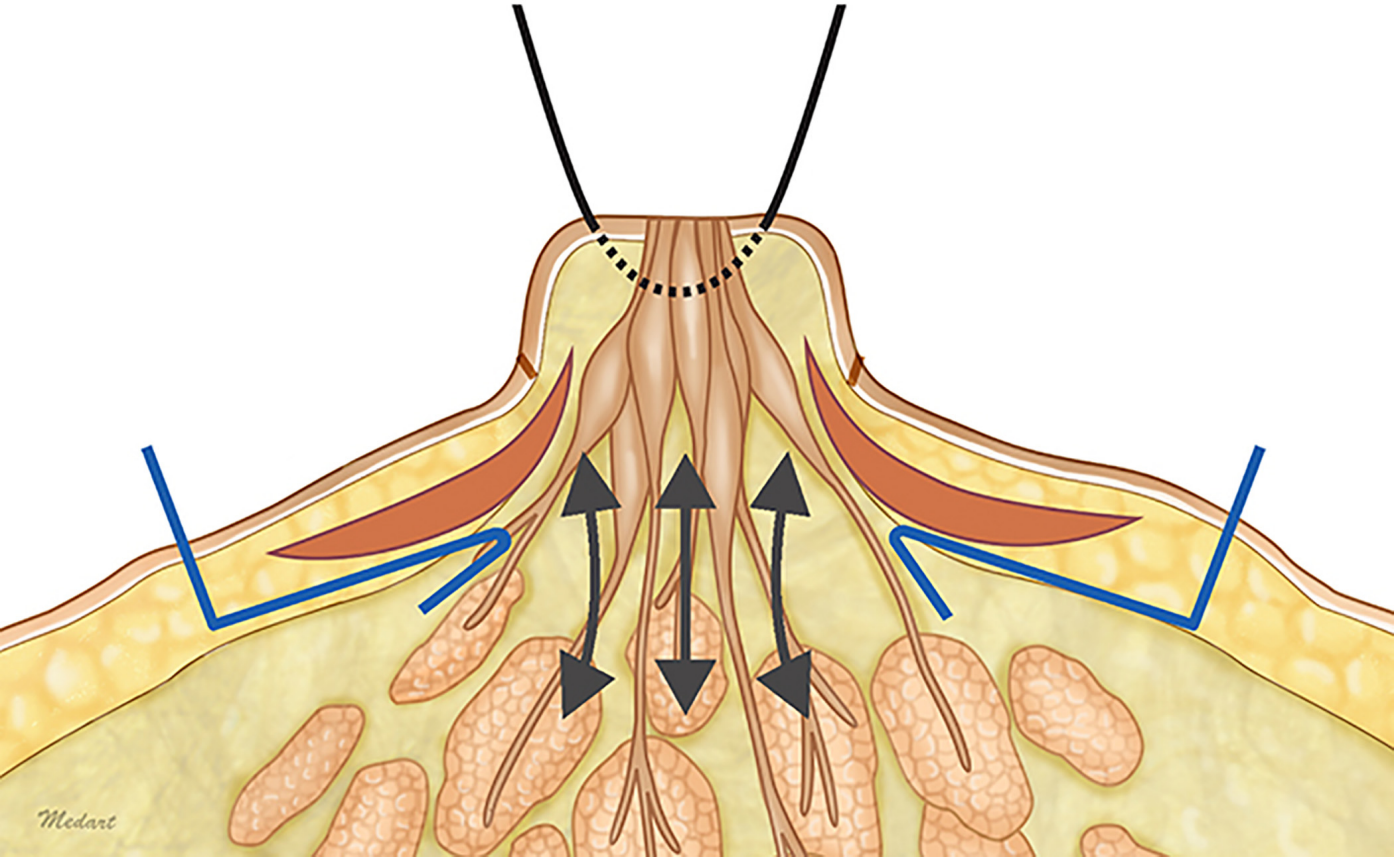
A schematic illustration of the bilateral de-epithelized subcutaneous triangular flaps. The triangular flaps, except for the bases, are incised down to the breast parenchyma (blue lines). Subcutaneous tunneling is performed below the level of the areolar floor by vertical blunt dissection (bidirectional arrows).

**Fig 3:**
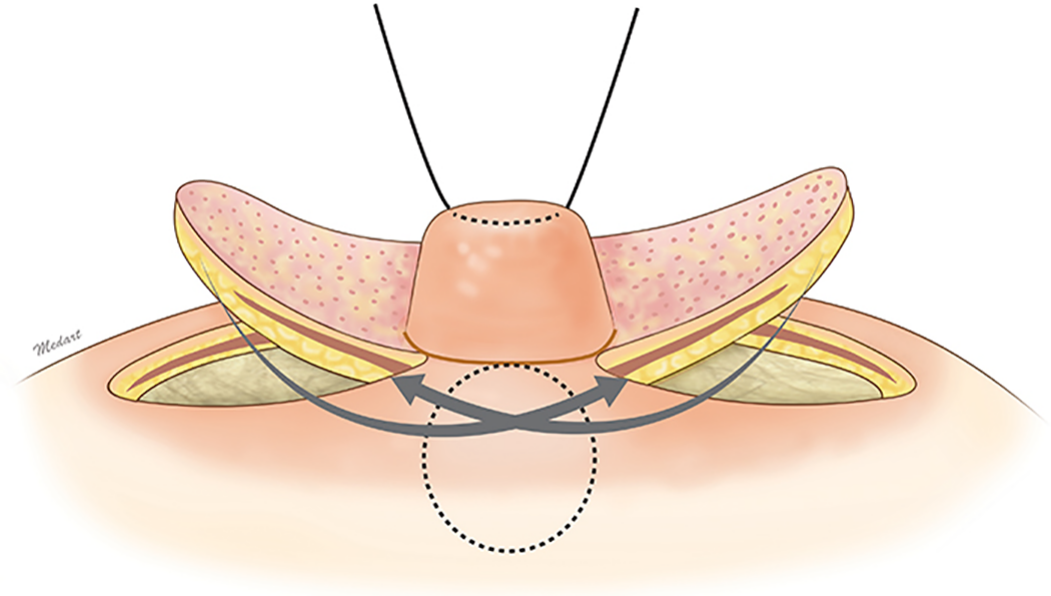
The triangular flaps are rotated 90 degrees and then crossed through the vertical slit. The red dotted line and the circular line at the nipple base are incised down to the upper dermis.

**Fig 4:**
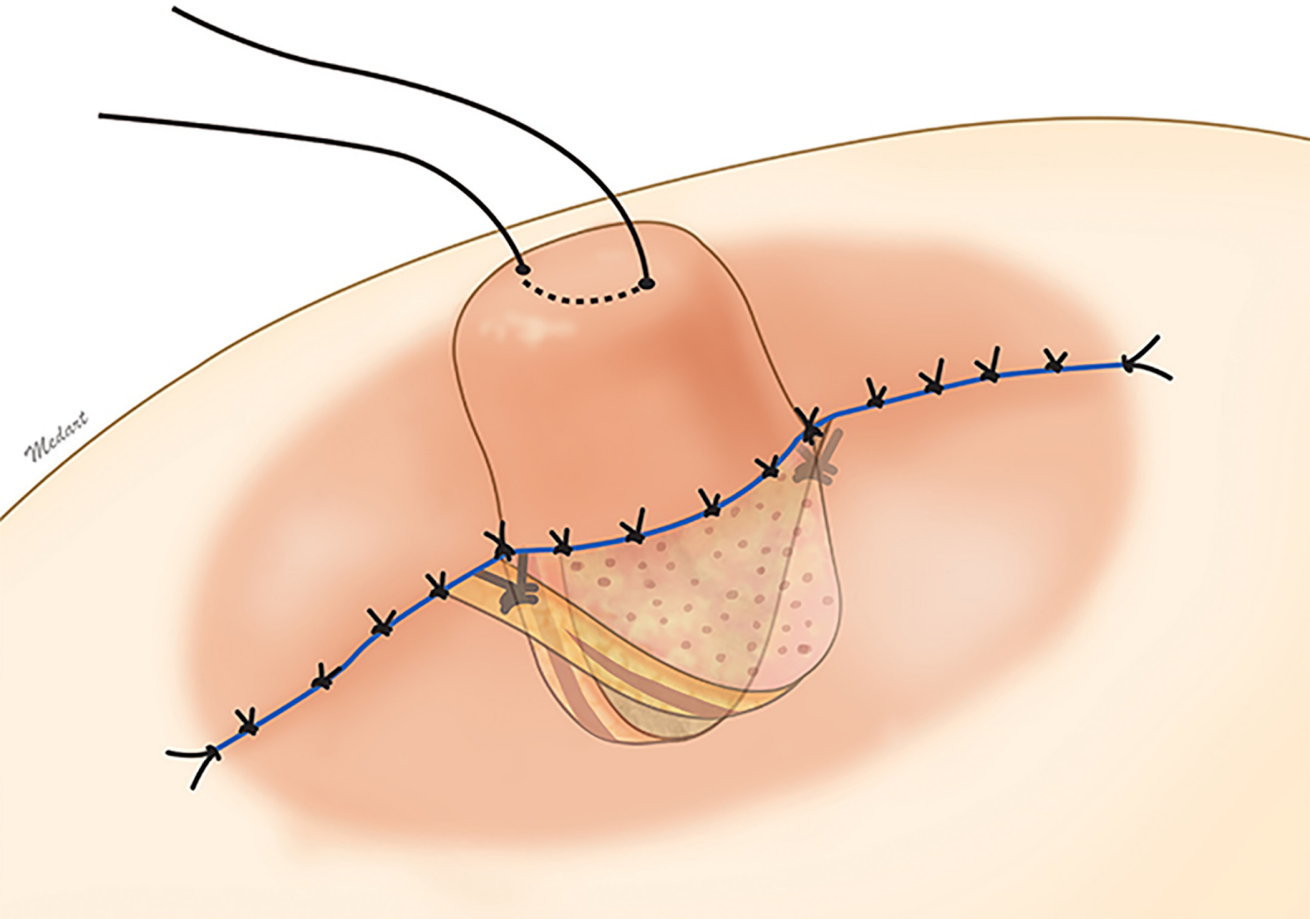
A schematic illustration of the postoperative view. Fixation is completed by filling the space under the nipple in the vertical direction. Re-draping of the nipple skin and surrounding areolar skin is also performed.

The protracted nipple was dressed in a light compressive dressing. The stay suture was maintained and the nipple was protected in a paper cup for a week. For two months following this, patients were instructed to wear one-cup larger brassiere along with a cotton stent made from a finger stockinet and Fixomull (BSN Medical, Hamburg, Germany)(Fig 5).

**Fig 5:**
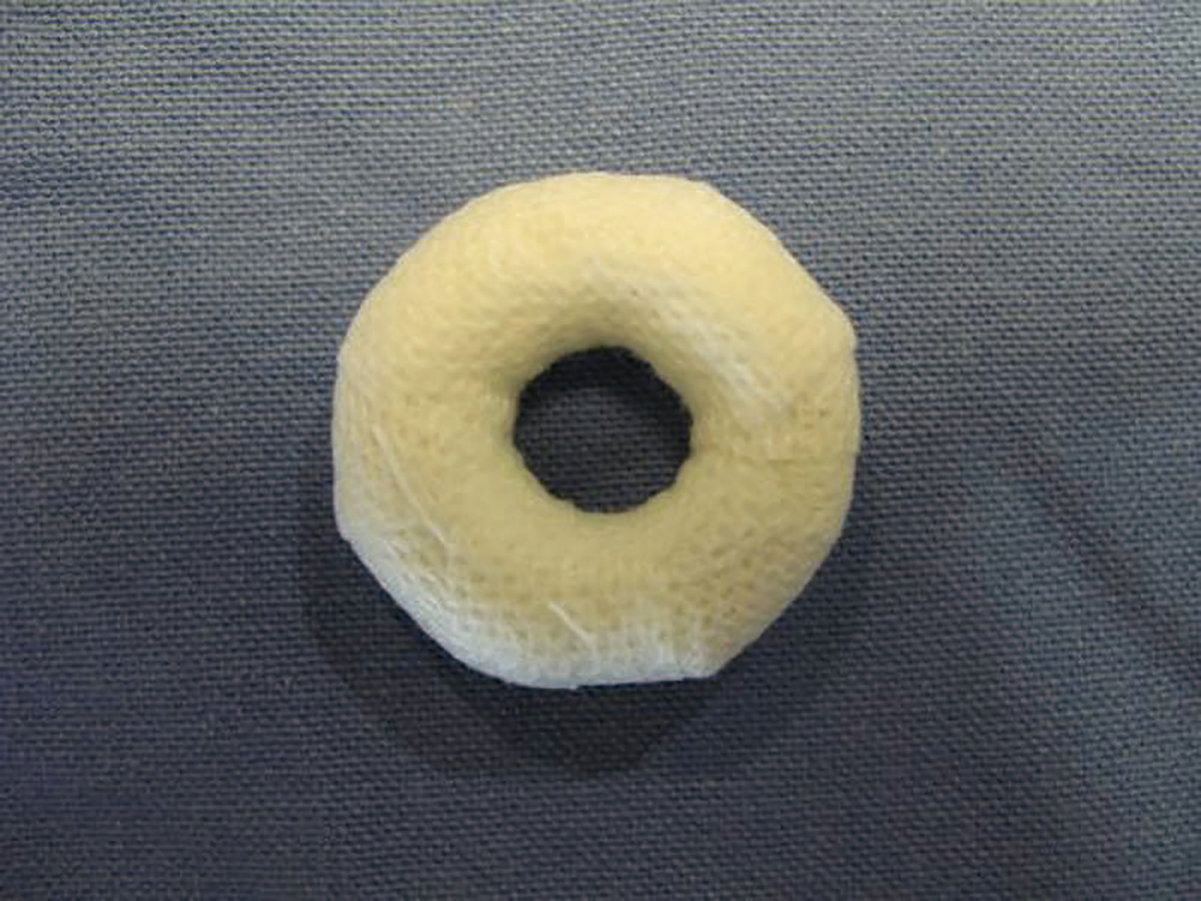
Postoperative care. A postoperative cotton stent made from a finger-rolled stockinet and hypoallergenic nonwoven adhesive tape (Fixomull, BSN Medical, Hamburg, Germany) is worn for two months.

## Results

The review identified 26 female patients among whom 47 inverted nipples were corrected using the method described above (Figs 6(A)–6(C), 7(A)–7(D) and 8(A)–8(B)). Twenty-one patients had inverted nipples bilaterally, and five patients had unilateral inverted nipples. Thirty-seven (37) of the nipples were grade II inversions, according to Han and Hong classification, with the remaining 10 nipples being grade III inversions [12].

**Fig 6:**
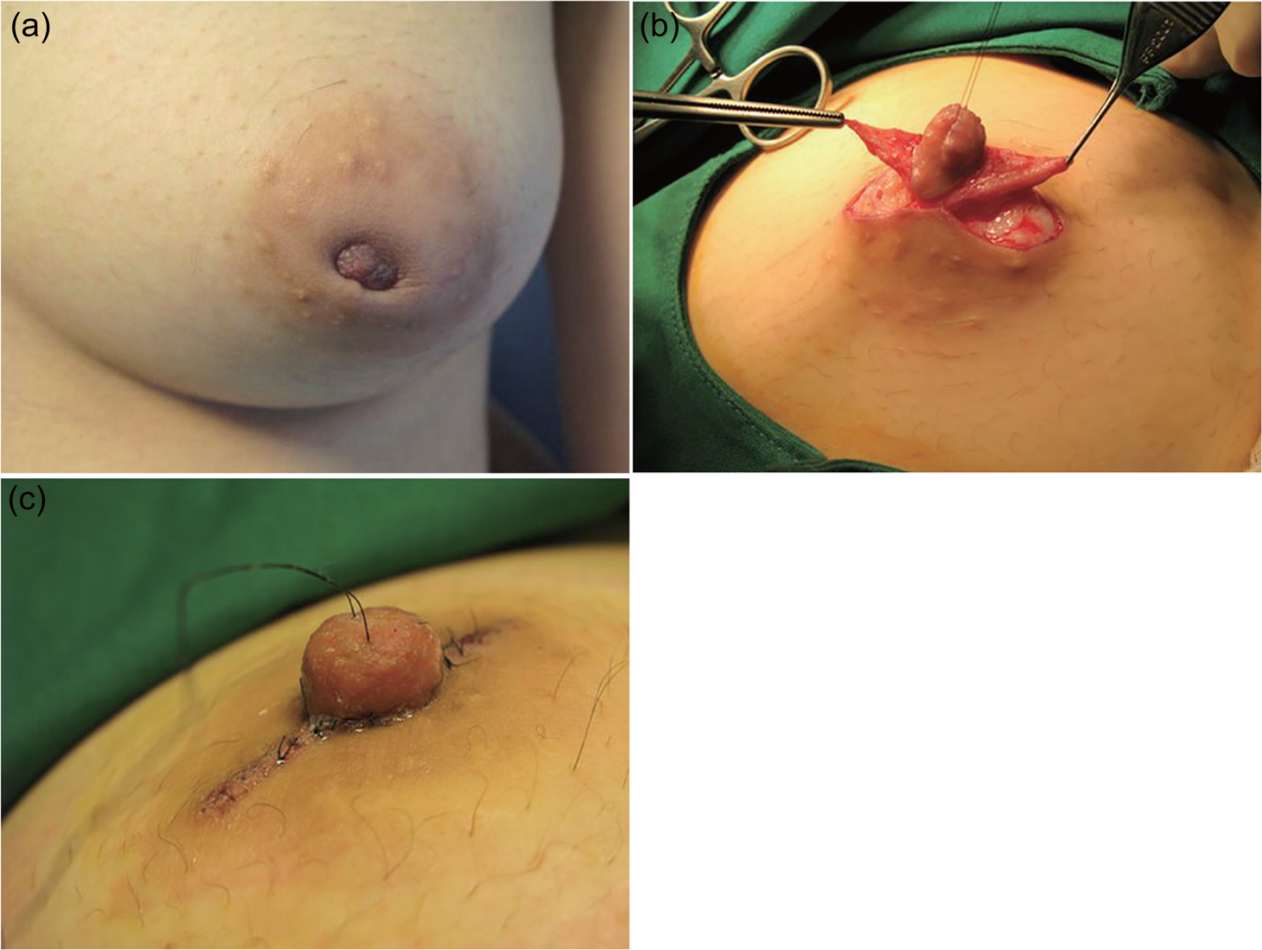
A 33-year-old patient with Grade II inversion. (a) Preoperative view of the left breast. (b) Intraoperative view of the de-epithelized triangular flaps. (c) Postoperative view at seven days.

**Fig 7:**
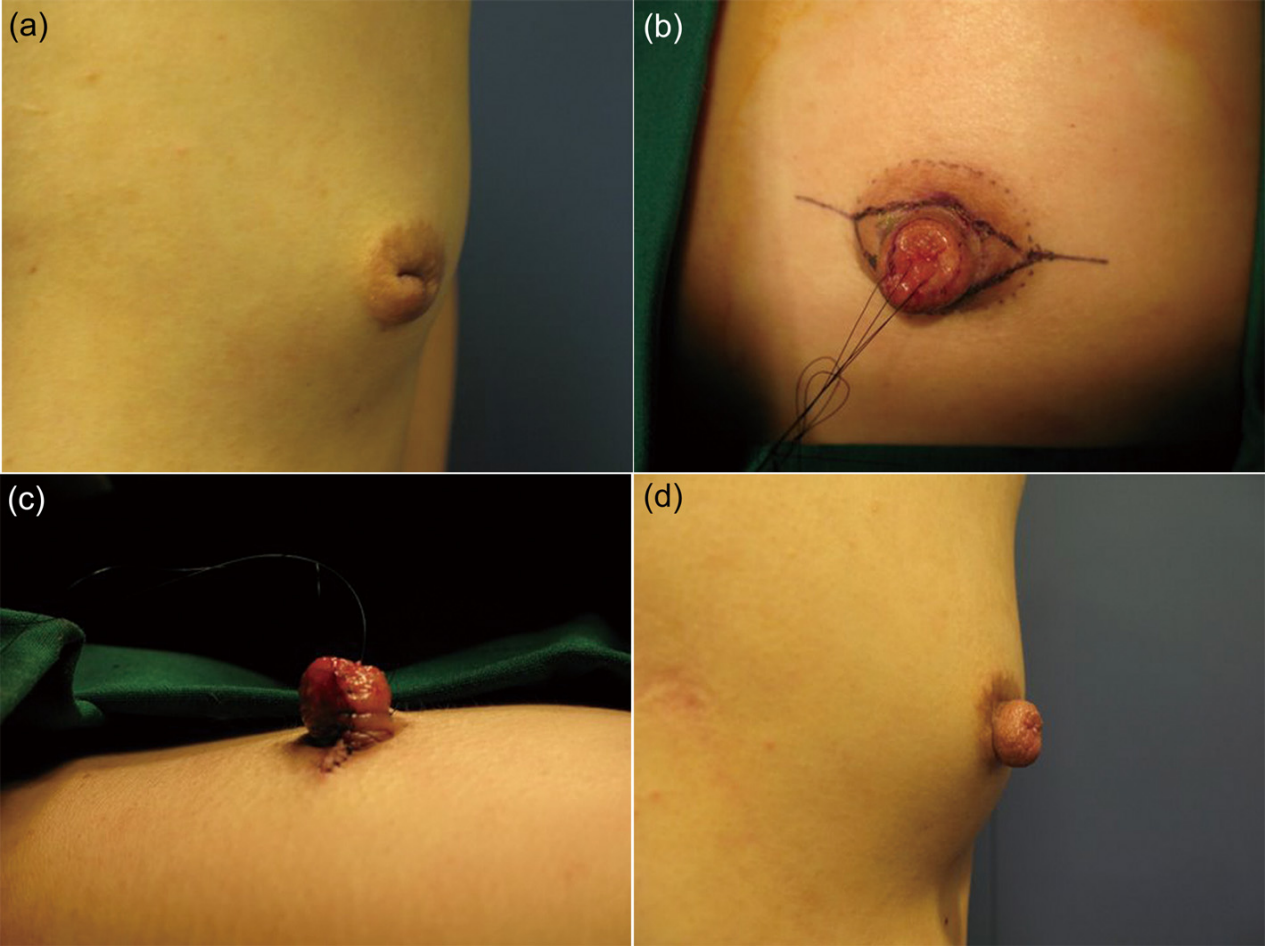
A 28-year-old patient with grade III inversion. (a) Preoperative view. (b, c) Surgical design and immediate postoperative view. (d) Postoperative result at seven months.

**Fig 8:**
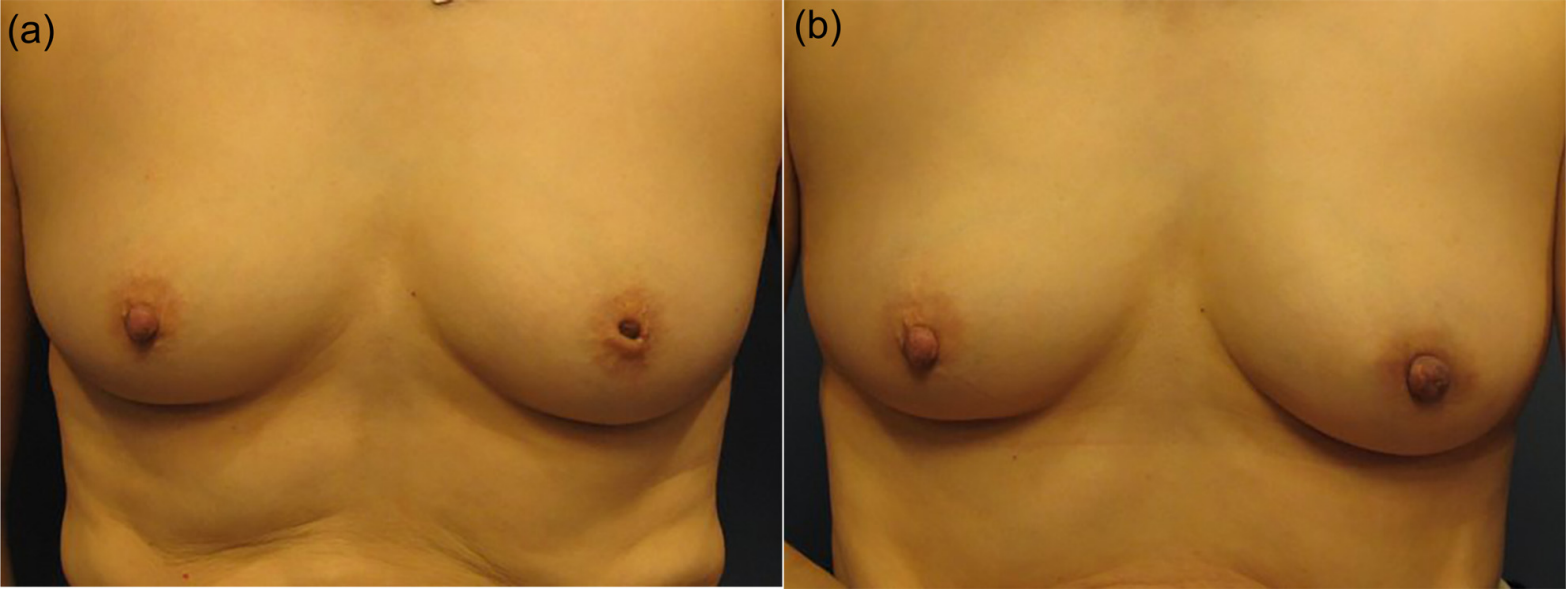
A 51-year-old patient with grade III inversion. (a) Preoperative view. (b) Postoperative result at 17 months.

The mean patient age was 34 years (range: 16–64 years) at time of operation. Twelve patients were planning the breastfeed in the future (n = 17; 45%). The remaining patients were neither breastfeeding nor planning to breastfeed (n = 9; 35%). Postoperative course was uneventful for all of the patients, and there were no recorded instances of wound infection, hematoma, or nipple necrosis.

Nipple height had been measured with calipers immediately after the operation and during routine clinic visits. The immediate post-op nipple height was 10.8 ± 0.8 mm, which had decreased to 9.0 ± 1.0 mm by the mean follow-up visit (14.03 months). In 45 of the cases, postoperative nipple projection remained at 90–100% of the nipple height achieved at the time of operation. In one patient, 40% of projection was lost in both nipples. All of the patients were satisfied with nipple contour and projection.

The brush test revealed that all 47 postoperative nipples had retained enough sensory function to elicit a contraction response [13]. Of the whole, five of the patients subsequently had children and were successful in breastfeeding.

## Discussion

Postoperative nipple height reductions have ranged from 10 to 50% for the purse-string suture method [14–16]. While procedures such as thick and full-thickness pennant flaps, dermoglandular flaps, or dermal elongation using a telescope at the base appear to be appropriate for correcting severely inverted nipples, recurrence rate remains significant for grade II and III inversions [14, 15, 17].

In certain techniques, the lactiferous duct is transected to correct severely inverted nipples [6,12, 17,18]. However, the nipple is innervated from the lateral cutaneous branches of the fourth intercostal nerve along the major duct system [19], and preserving the lactiferous duct is important not only for breastfeeding but also for nipple sensation.

In our series, the postoperative nipple height remained at 9 mm. Nearly all of the corrected nipples had maintained adequate nipple height, with the exception being one patient in whom the postoperative height had decreased by 40% in both nipples. Additionally, no inversion had recurred in any of the nipples.

Nipple sensation can be evaluated by a number of methods. The postoperative nipple has been examined most frequently by subjective patient report. Semmes-Weinstein monofilament test and pressure specified sensory device may have the advantage of quantifiable data [20]. However, such tests designed to measure pressure or dimensional sensitivity may not be as clinically significant in the assessment of nipple. Whereas tactile gnosis is important to the hand and pressure sensitivity is important to the foot, the relevant physiologic functions are sexual arousal, oxytocin secretion, and lactation for the nipple. One of the earliest clinical studies on nipple sensation has revealed erectility of the nipple upon stimulation was found to be most relevant to nipple function, with more objective methods of sensation assessment failing to account for what is physiologically relevant to a patient’s sexual and reproductive needs. [21, 22] Hence, we rely on the contractile response upon brush stimulation to most closely represent the ability generate either sexual or suckling response from the nipple. Thus far in our series of patients undergoing the modified method of nipple correction, all of the operated nipples had either retained or recovered the type of sensory perception required for the pilomotor-like response.

In review, we have summarized four of the most widely reported inverted nipple correction methods in comparison to our modified method (Table 1). Our method has four main differences to previous methods. The first modified component is that the bases of triangular flaps were rotated, downturned, and fixated such that the nipple base was mechanically cross-linked in the dermal plane defined by the areola mound. This procedure evenly disperses the fixation tension laterally and translates anterior-posterior vector laterally into the areola tissue. In contrast, Elsahy fixed the triangular flap tips to one side of the areolar plane by transposition, which does not provide similar mechanical linkage between the nipple base and the areola dermis [5].

**Table 1:** Various deepithelized triangular flaps for correction of inverted nipple^8^.

Methods	Elsahy [4]	Teimourian [6]	Kim [8]	Burm [9]	Lee (Current study)
***Direction of the flap***	Turned over	Turned down	Turned down	Turned over	Twisted and turned over
***Flap fixation***	Opposite areolar dermis overlapping	Each other under the nipple, not overlapping	Opposite dermis of nipple base, overlapping	Opposite areolar dermis, overlapping	Opposite areolar dermis, Vertically overlapping
***Position of flap*** *	Side	Beneath	Between	Between	Between
***Tenting effect***	Yes	No	No	Yes	Yes
***Components***	Dermal	Dermal	Dermal	Dermal	Subcutaneous
***Circumferential incision***	Yes	No	No	No	Yes
***Lactiferous ducts***	Preserve	Transect	Preserve	Preserve	Preserve
***Special postop care***	Yes	?	No	No	Yes

* **Location of the deepithelized triangular flaps to the side, beneath, or between the lactiferous ducts.**

? No explanation

The second component is that the longitudinal length of each triangular flap was equal to the diameter of nipple base, which length maintains tension and provides strong suspension after fixation.

The third component of our design is that the bases of triangular flaps are wider than that described by Elsahy. This base was approximately one quarter of the circumference of nipple base. This wider base had two purposes. The first was to create for a greater purse-string effect at the nipple base at the time of primary closure of the subcutaneous layer and areola skin. The second was to provide as reliable of a blood supply to the triangular flaps.

Our fourth modification is the adoption of thick and reliable subcutaneous flaps, which included the areolomammillary muscle layer. These downturned and buried flaps were designed to occupy the vertical tunnel just deep to the nipple base. The bulk of these flaps act as supporting pillars below the nipple, fill the dead space, and thereby discourage retraction of the nipple. Two methods incorporating thick subcutaneous flaps have previously been introduced. Hugo et al. used a double-opposing pennant flap, and Taylor et al. employed areola-based dermoglandular advancement using rhomboid flaps [14, 17]. These methods differ from our support and push-out efforts

We applied a circumferential incision around the nipple base. This thick subcutaneous flap is also complementary to the circumferential incision around the base, which is helpful for creating and maintaining a naturally symmetric and upright position for the nipple and making repositioning easier without compromising blood supply. This circumferential incision was an original design by Elsahy but is not widely adopted for the fear of violating the blood supply to the nipple [5–11, 13].

Postoperative care is also important in the prevention of recurring nipple inversion, and wearing a prefabricated cotton stent and a larger brassiere is a simple, tolerable solution for patients.

In conclusion, the suspension technique of crossing bulky subcutaneous triangular flaps with a circumferential dermal incision at the nipple base is a simple, reliable method for correcting nipple inversion.
